# Bis[4-amino-*N*-(pyrimidin-2-yl)benzene­sulfonamidato]-κ^2^ *N*,*N*′;κ*N*-aqua­bis­(dimethyl­formamide-κ*O*)cadmium(II) monohydrate

**DOI:** 10.1107/S1600536811019635

**Published:** 2011-05-28

**Authors:** G. M. Golzar Hossain

**Affiliations:** a Department of Chemistry, University of Dhaka, Dhaka 1000, Bangladesh

## Abstract

In the title compound, [Cd(C_10_H_9_N_4_O_2_S)_2_(C_3_H_7_NO)_2_(H_2_O)]·H_2_O, the Cd^II^ ion displays a grossly distorted octa­hedral (or irregular) *mer*-CdN_3_O_3_ coodination polyhdron arising from its coordination by one *N*,*N*′-bidentate sulfadiazinate anion, one monodentate sulfadiazinate anion, two O-bonded dimethyl­formamide molecules and one water mol­ecule. A short Cd⋯N contact [2.890 (3) Å] to the monodentate sulfadiazinate ion also occurs. The dihedral angles between the planes of the aromatic rings of the anions are 86.81 (14) and 68.65 (14)°. The crystal structure features inter­molecular N—H⋯O, O—H⋯O and O—H⋯N hydrogen bonds.

## Related literature

For the geometric analysis of related structures, see: Heren *et al.* (2006 ▶); Hossain & Amoroso (2007 ▶); Paşaoğlu *et al.* (2008 ▶); Hossain (2011 ▶).
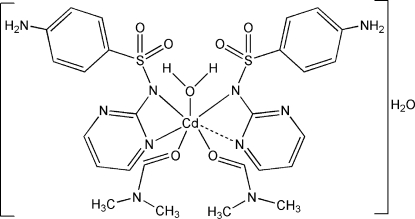


## Experimental

### 

#### Crystal data

[Cd(C_10_H_9_N_4_O_2_S)_2_(C_3_H_7_NO)_2_(H_2_O)]·H_2_O
*M* *_r_* = 793.17
Monoclinic, 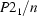

*a* = 13.9012 (3) Å
*b* = 9.9763 (2) Å
*c* = 23.8147 (5) Å
β = 103.203 (1)°
*V* = 3215.38 (12) Å^3^
*Z* = 4
Mo *K*α radiation
μ = 0.87 mm^−1^
*T* = 150 K
0.10 × 0.10 × 0.04 mm

#### Data collection

Nonius KappaCCD diffractometer
Absorption correction: multi-scan (*SORTAV*; Blessing, 1995 ▶) *T*_min_ = 0.918, *T*_max_ = 0.966
24302 measured reflections
7313 independent reflections
5720 reflections with *I* > 2σ(*I*)
*R*_int_ = 0.080

#### Refinement

*R*[*F* ^2^ > 2σ(*F* ^2^)] = 0.042
*wR*(*F* ^2^) = 0.099
*S* = 1.03
7313 reflections
460 parameters
12 restraints
H atoms treated by a mixture of independent and constrained refinement
Δρ_max_ = 0.63 e Å^−3^
Δρ_min_ = −1.06 e Å^−3^

### 

Data collection: *DENZO* (Otwinowski & Minor, 1997 ▶) and *COLLECT* (Hooft, 1998 ▶); cell refinement: *DENZO* and *COLLECT*; data reduction: *DENZO* and *COLLECT*; program(s) used to solve structure: *SHELXS97* (Sheldrick, 2008 ▶); program(s) used to refine structure: *SHELXL97* (Sheldrick, 2008 ▶); molecular graphics: *ORTEP-3 for Windows* (Farrugia, 1997 ▶); software used to prepare material for publication: *WinGX* (Farrugia, 1999 ▶).

## Supplementary Material

Crystal structure: contains datablocks I, global. DOI: 10.1107/S1600536811019635/hb5888sup1.cif

Structure factors: contains datablocks I. DOI: 10.1107/S1600536811019635/hb5888Isup2.hkl

Additional supplementary materials:  crystallographic information; 3D view; checkCIF report

## Figures and Tables

**Table 1:** Selected bond lengths (Å)

Cd1—O1	2.284 (2)
Cd1—O2	2.343 (2)
Cd1—O3	2.334 (2)
Cd1—N11	2.473 (2)
Cd1—N12	2.361 (2)
Cd1—N21	2.257 (2)
Cd1—N22	2.890 (3)

**Table 2:** Hydrogen-bond geometry (Å, °)

*D*—H⋯*A*	*D*—H	H⋯*A*	*D*⋯*A*	*D*—H⋯*A*
N14—H14*B*⋯O12^i^	0.82 (2)	2.39 (3)	3.104 (4)	146 (4)
N14—H14*A*⋯O21^ii^	0.81 (2)	2.47 (2)	3.267 (4)	167 (4)
N24—H24*B*⋯O4^iii^	0.84 (2)	2.27 (2)	3.061 (4)	157 (3)
N24—H24*A*⋯O3^iii^	0.79 (2)	2.52 (2)	3.272 (4)	160 (3)
O3—H3*E*⋯O11	0.79 (2)	1.97 (2)	2.754 (3)	172 (4)
O3—H3*D*⋯O11^iv^	0.81 (2)	2.04 (3)	2.810 (3)	159 (3)
O4—H4*B*⋯O21	0.85 (3)	2.02 (4)	2.855 (3)	166 (12)
O4—H4*A*⋯N23^v^	0.85 (3)	2.30 (5)	3.051 (4)	148 (7)

Symmetry codes: (i) 
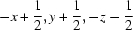
; (ii) 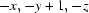; (iii) 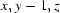; (iv) 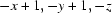; (v) 
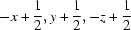
.

**Fig. 1.**
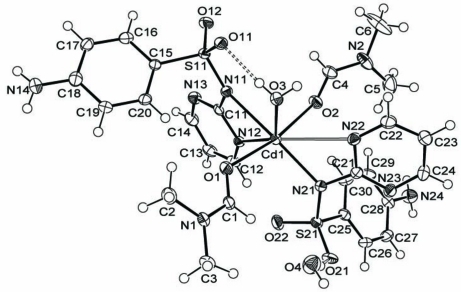
The molecular structure of (I), with 50% probability displacement ellipsoids for non-H atoms.

**Fig. 2.**
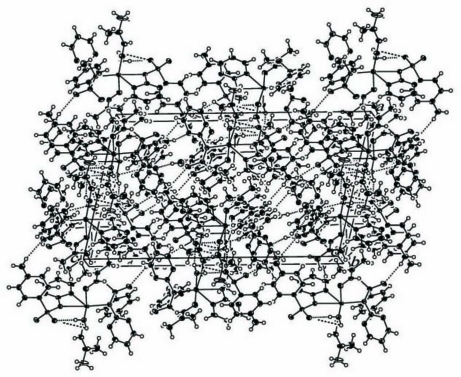
The packing of (I), viewed down the a-axis, showing one layer of molecules connected by N—H···N, N—H···O and O—H···O hydrogen bonds (dashed lines).

## supplementary crystallographic information

### Comment

The Cd ion in the title compound, (I), is attached with two sulfadiazine, two dimethylformamide and one water molecules. In addition there is one more solvated water molecule in the lattice. The Cd ion displays a hepta-coordinated complex but one of the pyrimido nitrogen atoms from the second sulfadiazinate anion has weak interaction with Cd(II) ion.

The hepta-coordinated cadmium complex is monocapped trigonal prismatic rather than the pentagonal bipyramidal structure as the angles around the cadmium centre are very much reduced from the ideal behavior since there exist different types of coordinated molecules.

The bond angles around the S atom correspond to a distorted tetrahedral geometry. The bond distance C—N (Terminal amino group) of 1.369 (4) and 1.375 (4)Å and the torsion angle C–S–N–C of 66.2 (3)° and -54.7 (3)° are comparable to those observed in the related structures (Heren *et al.*, 2006; Hossain & Amoroso, 2007; Hossain, 2011).

The dihedral angle between the aromatic rings of the chelated anion of 88.82 (7)° is larger than value of 68.66 (9)° of the non-chelated anion. The later one is comparable with the value of 71.10 (14)° (Hossain & Amoroso, 2007) in the sulfadiazinate anion. The packing of (I) (Fig. 2) is stabilized by intermolecular N—H···O, O—H···O and O—H···N hydrogen bonds (Table 2) and exists among sdz anions, coordinated and solvated water molecules (Hossain, 2011).

### Experimental

The sodium salt of sulfadiazine (Nasdz, 5.446 g, 2 mmol) was dissolved in hot methanol (50 ml) and a methanol solution (10 ml) of (CH_3_COO)_2_Cd.2H_2_O (2.6647 g, 1 mmol) was added slowly with constant stirring on a hot plate. A white precipitate was formed and the mixture was stirred for a further 2 h. The precipitate was filtered off and dried over silica gel; it was then dissolved in dimethylformamide solution (50 ml), and the mixture stirred for 10 min., filtered and left for crystallisaton. A week later, white block-shaped crystals of (I) were filtered off and dried over silica gel.

### Refinement

The H atoms were positioned geometrically and refined using a riding model [except that for terminal amino groups N14 and N24 and water molecules which were located from the difference map and fixed to 0.79 (2)–0.85 (3) Å], with C—H = 0.95–0.98 Å and with *U*_iso_(H) = 1.2 (1.5 for methyl groups) times *U*_eq_(C).

### Crystal data

**Table:**

[Cd(C_10_H_9_N_4_O_2_S)_2_(C_3_H_7_NO)_2_(H_2_O)]·H_2_O	*F*(000) = 1624
*M**_r_* = 793.17	*D*_x_ = 1.638 Mg m^−^^3^
Monoclinic, *P*2_1_/*n*	Mo *K*α radiation, λ = 0.71073 Å
Hall symbol: -P 2yn	Cell parameters from 7313 reflections
*a* = 13.9012 (3) Å	θ = 2.9–27.5°
*b* = 9.9763 (2) Å	µ = 0.87 mm^−^^1^
*c* = 23.8147 (5) Å	*T* = 150 K
β = 103.203 (1)°	Block, colourless
*V* = 3215.38 (12) Å^3^	0.10 × 0.10 × 0.04 mm
*Z* = 4	

### Data collection

**Table:**

Nonius KappaCCD diffractometer	7313 independent reflections
Radiation source: fine-focus sealed tube	5720 reflections with *I* > 2σ(*I*)
graphite	*R*_int_ = 0.080
ω scans	θ_max_ = 27.5°, θ_min_ = 3.1°
Absorption correction: multi-scan (*SORTAV*; Blessing, 1995)	*h* = −18→17
*T*_min_ = 0.918, *T*_max_ = 0.966	*k* = −12→12
24302 measured reflections	*l* = −30→30

### Refinement

**Table:**

Refinement on *F*^2^	Primary atom site location: structure-invariant direct methods
Least-squares matrix: full	Secondary atom site location: difference Fourier map
*R*[*F*^2^ > 2σ(*F*^2^)] = 0.042	Hydrogen site location: inferred from neighbouring sites
*wR*(*F*^2^) = 0.099	H atoms treated by a mixture of independent and constrained refinement
*S* = 1.03	*w* = 1/[σ^2^(*F*_o_^2^) + (0.0412*P*)^2^ + 1.8016*P*] where *P* = (*F*_o_^2^ + 2*F*_c_^2^)/3
7313 reflections	(Δ/σ)_max_ = 0.001
460 parameters	Δρ_max_ = 0.63 e Å^−^^3^
12 restraints	Δρ_min_ = −1.06 e Å^−^^3^

### Special details

**Table:**

Geometry. All e.s.d.'s (except the e.s.d. in the dihedral angle between two l.s. planes) are estimated using the full covariance matrix. The cell e.s.d.'s are taken into account individually in the estimation of e.s.d.'s in distances, angles and torsion angles; correlations between e.s.d.'s in cell parameters are only used when they are defined by crystal symmetry. An approximate (isotropic) treatment of cell e.s.d.'s is used for estimating e.s.d.'s involving l.s. planes.
Refinement. Refinement of *F*^2^ against ALL reflections. The weighted *R*-factor *wR* and goodness of fit *S* are based on *F*^2^, conventional *R*-factors *R* are based on *F*, with *F* set to zero for negative *F*^2^. The threshold expression of *F*^2^ > σ(*F*^2^) is used only for calculating *R*-factors(gt) *etc*. and is not relevant to the choice of reflections for refinement. *R*-factors based on *F*^2^ are statistically about twice as large as those based on *F*, and *R*- factors based on ALL data will be even larger.

### Fractional atomic coordinates and isotropic or equivalent isotropic displacement parameters (Å^2^)

**Table:**

	*x*	*y*	*z*	*U*_iso_*/*U*_eq_	
Cd1	0.290291 (16)	0.29728 (2)	0.034509 (9)	0.01727 (8)	
S11	0.33422 (5)	0.41881 (7)	−0.11138 (3)	0.01623 (16)	
S21	0.18412 (5)	0.12716 (7)	0.12871 (3)	0.01808 (17)	
O11	0.39815 (15)	0.5141 (2)	−0.07301 (9)	0.0205 (5)	
O12	0.38338 (16)	0.3485 (2)	−0.14979 (9)	0.0240 (5)	
O21	0.15043 (16)	0.1606 (2)	0.18039 (9)	0.0238 (5)	
O22	0.11094 (16)	0.1429 (2)	0.07524 (9)	0.0272 (5)	
N11	0.29064 (18)	0.3272 (2)	−0.06853 (10)	0.0167 (5)	
N12	0.19324 (18)	0.1714 (2)	−0.04169 (10)	0.0174 (5)	
N13	0.1916 (2)	0.1903 (2)	−0.14243 (11)	0.0216 (6)	
N14	0.0348 (2)	0.7936 (3)	−0.24158 (14)	0.0330 (7)	
N21	0.27561 (18)	0.2170 (2)	0.12076 (10)	0.0176 (5)	
N22	0.43358 (19)	0.2942 (2)	0.14358 (11)	0.0197 (6)	
N23	0.38318 (19)	0.1370 (2)	0.20728 (10)	0.0208 (6)	
N24	0.3315 (2)	−0.4299 (3)	0.16052 (14)	0.0304 (7)	
C11	0.2235 (2)	0.2273 (3)	−0.08703 (12)	0.0169 (6)	
C12	0.1256 (2)	0.0745 (3)	−0.05344 (14)	0.0221 (7)	
H12	0.1024	0.0350	−0.0227	0.027*	
C13	0.0887 (2)	0.0302 (3)	−0.10880 (14)	0.0247 (7)	
H13	0.0408	−0.0392	−0.1171	0.030*	
C14	0.1249 (2)	0.0914 (3)	−0.15177 (14)	0.0251 (7)	
H14	0.1009	0.0615	−0.1903	0.030*	
C15	0.2381 (2)	0.5159 (3)	−0.15305 (12)	0.0162 (6)	
C16	0.2380 (2)	0.5497 (3)	−0.20973 (13)	0.0210 (7)	
H16	0.2844	0.5095	−0.2283	0.025*	
C17	0.1704 (2)	0.6418 (3)	−0.23916 (13)	0.0229 (7)	
H17	0.1718	0.6655	−0.2776	0.028*	
C18	0.1002 (2)	0.7003 (3)	−0.21307 (14)	0.0231 (7)	
C19	0.0994 (2)	0.6630 (3)	−0.15630 (13)	0.0218 (7)	
H19	0.0509	0.6995	−0.1383	0.026*	
C20	0.1687 (2)	0.5733 (3)	−0.12621 (13)	0.0190 (6)	
H20	0.1689	0.5510	−0.0874	0.023*	
C21	0.3658 (2)	0.2140 (3)	0.15960 (12)	0.0169 (6)	
C22	0.5243 (2)	0.2924 (3)	0.17670 (14)	0.0256 (7)	
H22	0.5734	0.3475	0.1665	0.031*	
C23	0.5504 (3)	0.2135 (3)	0.22563 (14)	0.0261 (7)	
H23	0.6159	0.2116	0.2486	0.031*	
C24	0.4763 (2)	0.1382 (3)	0.23904 (13)	0.0252 (7)	
H24	0.4918	0.0838	0.2726	0.030*	
C25	0.2238 (2)	−0.0401 (3)	0.13588 (12)	0.0181 (6)	
C26	0.1971 (2)	−0.1222 (3)	0.17657 (13)	0.0211 (7)	
H26	0.1548	−0.0888	0.1995	0.025*	
C27	0.2315 (2)	−0.2522 (3)	0.18403 (13)	0.0227 (7)	
H27	0.2123	−0.3081	0.2119	0.027*	
C28	0.2945 (2)	−0.3021 (3)	0.15076 (14)	0.0207 (7)	
C29	0.3201 (2)	−0.2190 (3)	0.10946 (15)	0.0259 (7)	
H29	0.3615	−0.2523	0.0860	0.031*	
C30	0.2860 (2)	−0.0889 (3)	0.10219 (13)	0.0240 (7)	
H30	0.3048	−0.0327	0.0743	0.029*	
C1	0.1274 (2)	0.4958 (3)	0.05636 (13)	0.0192 (6)	
H1	0.1262	0.4329	0.0862	0.023*	
C2	0.0778 (3)	0.7114 (3)	0.01313 (16)	0.0313 (8)	
H2A	0.1256	0.6877	−0.0098	0.047*	
H2B	0.0968	0.7970	0.0328	0.047*	
H2C	0.0119	0.7198	−0.0123	0.047*	
C3	0.0160 (2)	0.6297 (3)	0.09716 (14)	0.0282 (7)	
H3A	0.0330	0.7164	0.1161	0.042*	
H3B	0.0281	0.5582	0.1262	0.042*	
H3C	−0.0539	0.6295	0.0771	0.042*	
C4	0.4966 (2)	0.1672 (3)	0.03565 (14)	0.0245 (7)	
H4	0.5077	0.2442	0.0145	0.029*	
C5	0.5645 (3)	−0.0106 (4)	0.10096 (16)	0.0415 (10)	
H5A	0.4949	−0.0214	0.1022	0.062*	
H5B	0.6044	0.0023	0.1402	0.062*	
H5C	0.5870	−0.0909	0.0841	0.062*	
C6	0.6737 (3)	0.1498 (4)	0.0652 (2)	0.0611 (14)	
H6A	0.6695	0.2336	0.0433	0.092*	
H6B	0.7068	0.0814	0.0469	0.092*	
H6C	0.7115	0.1651	0.1048	0.092*	
N1	0.07654 (18)	0.6072 (2)	0.05576 (10)	0.0199 (5)	
N2	0.5748 (2)	0.1044 (3)	0.06631 (13)	0.0310 (7)	
O1	0.17698 (16)	0.4670 (2)	0.02063 (9)	0.0246 (5)	
O2	0.40965 (16)	0.1327 (2)	0.03247 (9)	0.0232 (5)	
O3	0.39437 (17)	0.4831 (2)	0.04135 (10)	0.0223 (5)	
O4	0.1616 (2)	0.4417 (3)	0.20345 (11)	0.0363 (6)	
H14A	−0.016 (2)	0.811 (4)	−0.2316 (16)	0.049 (14)*	
H14B	0.030 (3)	0.812 (4)	−0.2758 (11)	0.044 (13)*	
H24A	0.355 (2)	−0.465 (3)	0.1372 (13)	0.031 (11)*	
H24B	0.295 (2)	−0.485 (3)	0.1728 (13)	0.020 (9)*	
H4A	0.157 (6)	0.467 (7)	0.2366 (18)	0.15 (3)*	
H4B	0.157 (15)	0.356 (3)	0.202 (6)	0.50 (12)*	
H3D	0.451 (2)	0.478 (4)	0.0587 (13)	0.040 (12)*	
H3E	0.394 (3)	0.499 (4)	0.0086 (10)	0.041 (12)*	

### Atomic displacement parameters (Å^2^)

**Table:**

	*U*^11^	*U*^22^	*U*^33^	*U*^12^	*U*^13^	*U*^23^
Cd1	0.01636 (13)	0.01826 (13)	0.01795 (13)	0.00356 (8)	0.00549 (9)	0.00285 (8)
S11	0.0137 (4)	0.0187 (4)	0.0166 (4)	−0.0014 (3)	0.0041 (3)	0.0005 (3)
S21	0.0150 (4)	0.0191 (4)	0.0207 (4)	0.0016 (3)	0.0053 (3)	0.0018 (3)
O11	0.0167 (11)	0.0240 (11)	0.0202 (11)	−0.0053 (9)	0.0030 (9)	0.0005 (8)
O12	0.0208 (12)	0.0300 (12)	0.0234 (12)	0.0021 (10)	0.0096 (10)	−0.0010 (9)
O21	0.0195 (12)	0.0241 (11)	0.0318 (12)	0.0025 (9)	0.0141 (10)	−0.0010 (9)
O22	0.0175 (12)	0.0323 (12)	0.0290 (12)	0.0007 (10)	−0.0007 (10)	0.0057 (10)
N11	0.0156 (13)	0.0190 (12)	0.0155 (12)	−0.0018 (10)	0.0033 (10)	−0.0005 (9)
N12	0.0149 (13)	0.0171 (12)	0.0212 (13)	0.0032 (10)	0.0063 (11)	0.0046 (10)
N13	0.0190 (14)	0.0239 (14)	0.0212 (14)	−0.0031 (11)	0.0034 (11)	−0.0034 (10)
N14	0.0244 (18)	0.0426 (19)	0.0319 (18)	0.0101 (14)	0.0061 (14)	0.0136 (14)
N21	0.0151 (13)	0.0211 (13)	0.0169 (13)	−0.0002 (10)	0.0043 (11)	0.0009 (10)
N22	0.0204 (14)	0.0206 (13)	0.0185 (13)	−0.0029 (10)	0.0051 (11)	0.0017 (10)
N23	0.0212 (14)	0.0223 (13)	0.0188 (13)	−0.0014 (11)	0.0043 (11)	0.0018 (10)
N24	0.0348 (18)	0.0231 (15)	0.0359 (18)	0.0035 (13)	0.0137 (15)	−0.0022 (13)
C11	0.0123 (15)	0.0188 (15)	0.0191 (15)	0.0054 (12)	0.0027 (12)	0.0008 (11)
C12	0.0175 (16)	0.0145 (15)	0.0370 (19)	0.0036 (12)	0.0119 (14)	0.0054 (13)
C13	0.0163 (17)	0.0195 (16)	0.0385 (19)	−0.0033 (13)	0.0067 (15)	−0.0033 (13)
C14	0.0196 (17)	0.0263 (17)	0.0276 (17)	−0.0031 (13)	0.0017 (14)	−0.0060 (13)
C15	0.0131 (15)	0.0171 (14)	0.0182 (15)	−0.0031 (11)	0.0030 (12)	0.0016 (11)
C16	0.0199 (17)	0.0241 (16)	0.0201 (15)	−0.0040 (13)	0.0070 (13)	−0.0018 (12)
C17	0.0244 (18)	0.0271 (17)	0.0171 (15)	−0.0023 (14)	0.0042 (14)	0.0026 (12)
C18	0.0211 (17)	0.0217 (16)	0.0242 (17)	−0.0053 (13)	0.0005 (14)	0.0027 (12)
C19	0.0175 (16)	0.0259 (16)	0.0235 (16)	0.0012 (13)	0.0078 (13)	0.0011 (13)
C20	0.0162 (16)	0.0213 (15)	0.0197 (15)	−0.0048 (12)	0.0045 (13)	0.0026 (12)
C21	0.0199 (16)	0.0146 (14)	0.0172 (15)	0.0032 (12)	0.0061 (13)	−0.0009 (11)
C22	0.0233 (18)	0.0260 (17)	0.0286 (18)	−0.0065 (13)	0.0081 (15)	0.0021 (13)
C23	0.0212 (18)	0.0300 (18)	0.0259 (17)	−0.0026 (14)	0.0029 (14)	−0.0010 (13)
C24	0.0275 (19)	0.0274 (17)	0.0180 (16)	0.0005 (14)	−0.0002 (14)	0.0029 (13)
C25	0.0147 (15)	0.0188 (15)	0.0201 (15)	−0.0010 (12)	0.0030 (13)	−0.0039 (12)
C26	0.0198 (17)	0.0240 (16)	0.0221 (16)	−0.0020 (13)	0.0104 (13)	−0.0018 (12)
C27	0.0253 (18)	0.0228 (15)	0.0214 (16)	−0.0033 (14)	0.0080 (14)	0.0032 (13)
C28	0.0175 (16)	0.0169 (15)	0.0264 (17)	−0.0006 (12)	0.0025 (13)	−0.0045 (12)
C29	0.0270 (19)	0.0203 (16)	0.0350 (19)	−0.0009 (13)	0.0166 (15)	−0.0055 (13)
C30	0.0270 (18)	0.0237 (16)	0.0243 (16)	−0.0041 (13)	0.0123 (14)	0.0018 (13)
C1	0.0149 (15)	0.0208 (15)	0.0210 (16)	0.0015 (12)	0.0019 (13)	0.0030 (12)
C2	0.031 (2)	0.0256 (18)	0.039 (2)	0.0124 (14)	0.0120 (17)	0.0102 (14)
C3	0.0226 (18)	0.0338 (19)	0.0301 (18)	0.0086 (14)	0.0099 (15)	0.0015 (14)
C4	0.0198 (18)	0.0236 (16)	0.0318 (18)	0.0069 (14)	0.0096 (15)	−0.0019 (13)
C5	0.041 (2)	0.042 (2)	0.038 (2)	0.0222 (18)	0.0033 (18)	0.0071 (17)
C6	0.017 (2)	0.047 (2)	0.117 (4)	0.0050 (18)	0.012 (2)	−0.016 (3)
N1	0.0162 (14)	0.0206 (13)	0.0216 (13)	0.0038 (10)	0.0017 (11)	0.0006 (10)
N2	0.0184 (15)	0.0265 (15)	0.0456 (18)	0.0070 (12)	0.0022 (13)	−0.0086 (13)
O1	0.0235 (12)	0.0270 (12)	0.0243 (11)	0.0100 (9)	0.0077 (10)	0.0027 (9)
O2	0.0184 (12)	0.0225 (11)	0.0291 (12)	0.0036 (9)	0.0064 (10)	0.0012 (9)
O3	0.0174 (13)	0.0292 (12)	0.0191 (12)	−0.0008 (10)	0.0020 (10)	0.0017 (10)
O4	0.0427 (16)	0.0327 (14)	0.0338 (15)	0.0031 (12)	0.0094 (13)	0.0016 (11)

### Geometric parameters (Å, °)

**Table:**

Cd1—O1	2.284 (2)	C19—H19	0.9500
Cd1—O2	2.343 (2)	C20—H20	0.9500
Cd1—O3	2.334 (2)	C22—C23	1.385 (5)
Cd1—N11	2.473 (2)	C22—H22	0.9500
Cd1—N12	2.361 (2)	C23—C24	1.369 (5)
Cd1—N21	2.257 (2)	C23—H23	0.9500
Cd1—N22	2.890 (3)	C24—H24	0.9500
S11—O11	1.469 (2)	C25—C26	1.383 (4)
S11—O12	1.443 (2)	C25—C30	1.394 (4)
S11—N11	1.589 (2)	C26—C27	1.379 (4)
S11—C15	1.762 (3)	C26—H26	0.9500
S21—O21	1.452 (2)	C27—C28	1.400 (4)
S21—O22	1.445 (2)	C27—H27	0.9500
S21—N21	1.602 (3)	C28—C29	1.393 (4)
S21—C25	1.754 (3)	C29—C30	1.379 (4)
N11—C11	1.368 (4)	C29—H29	0.9500
N12—C12	1.332 (4)	C30—H30	0.9500
N12—C11	1.364 (4)	C1—O1	1.245 (3)
N13—C14	1.337 (4)	C1—N1	1.316 (4)
N13—C11	1.344 (4)	C1—H1	0.9500
N14—C18	1.369 (4)	C2—N1	1.456 (4)
N14—H14A	0.81 (2)	C2—H2A	0.9800
N14—H14B	0.82 (2)	C2—H2B	0.9800
N21—C21	1.379 (4)	C2—H2C	0.9800
N22—C22	1.326 (4)	C3—N1	1.452 (4)
N22—C21	1.355 (4)	C3—H3A	0.9800
N23—C24	1.342 (4)	C3—H3B	0.9800
N23—C21	1.346 (4)	C3—H3C	0.9800
N24—C28	1.375 (4)	C4—O2	1.243 (4)
N24—H24A	0.79 (2)	C4—N2	1.320 (4)
N24—H24B	0.84 (2)	C4—H4	0.9500
C12—C13	1.374 (5)	C5—N2	1.439 (5)
C12—H12	0.9500	C5—H5A	0.9800
C13—C14	1.381 (4)	C5—H5B	0.9800
C13—H13	0.9500	C5—H5C	0.9800
C14—H14	0.9500	C6—N2	1.454 (5)
C15—C16	1.391 (4)	C6—H6A	0.9800
C15—C20	1.396 (4)	C6—H6B	0.9800
C16—C17	1.385 (4)	C6—H6C	0.9800
C16—H16	0.9500	O3—H3D	0.81 (2)
C17—C18	1.399 (5)	O3—H3E	0.79 (2)
C17—H17	0.9500	O4—H4A	0.85 (3)
C18—C19	1.405 (4)	O4—H4B	0.85 (3)
C19—C20	1.388 (4)		
			
N11—Cd1—N12	55.37 (8)	C20—C19—H19	119.7
N11—Cd1—N21	165.24 (8)	C18—C19—H19	119.7
N11—Cd1—N22	137.39 (8)	C19—C20—C15	119.9 (3)
N11—Cd1—O1	85.67 (8)	C19—C20—H20	120.0
N11—Cd1—O2	84.38 (8)	C15—C20—H20	120.0
N11—Cd1—O3	80.31 (8)	N23—C21—N22	124.9 (3)
N12—Cd1—N21	110.87 (8)	N23—C21—N21	122.6 (3)
N12—Cd1—N22	147.24 (7)	N22—C21—N21	112.5 (2)
N12—Cd1—O1	91.66 (8)	N22—C22—C23	122.6 (3)
N12—Cd1—O2	83.93 (8)	N22—C22—H22	118.7
N12—Cd1—O3	135.44 (8)	C23—C22—H22	118.7
N21—Cd1—N22	50.60 (8)	C24—C23—C22	116.3 (3)
N21—Cd1—O1	101.06 (8)	C24—C23—H23	121.9
N21—Cd1—O2	88.97 (8)	C22—C23—H23	121.9
N21—Cd1—O3	113.68 (8)	N23—C24—C23	123.6 (3)
N22—Cd1—O1	116.67 (7)	N23—C24—H24	118.2
N22—Cd1—O2	70.44 (7)	C23—C24—H24	118.2
N22—Cd1—O3	69.92 (7)	C26—C25—C30	119.9 (3)
O1—Cd1—O2	169.94 (7)	C26—C25—S21	120.2 (2)
O1—Cd1—O3	79.49 (8)	C30—C25—S21	119.9 (2)
O2—Cd1—O3	97.32 (8)	C27—C26—C25	120.4 (3)
O11—S11—O12	113.72 (13)	C27—C26—H26	119.8
O12—S11—N11	115.62 (13)	C25—C26—H26	119.8
O11—S11—N11	103.77 (12)	C26—C27—C28	120.3 (3)
O11—S11—C15	105.54 (13)	C26—C27—H27	119.9
O12—S11—C15	108.57 (13)	C28—C27—H27	119.9
N11—S11—C15	109.06 (13)	N24—C28—C29	121.7 (3)
O21—S21—O22	115.05 (13)	N24—C28—C27	119.5 (3)
O22—S21—N21	104.66 (13)	C29—C28—C27	118.7 (3)
O21—S21—N21	112.96 (13)	C30—C29—C28	120.9 (3)
O21—S21—C25	106.93 (13)	C30—C29—H29	119.6
O22—S21—C25	109.36 (13)	C28—C29—H29	119.6
N21—S21—C25	107.66 (13)	C29—C30—C25	119.8 (3)
C11—N11—S11	123.0 (2)	C29—C30—H30	120.1
C11—N11—Cd1	94.35 (17)	C25—C30—H30	120.1
S11—N11—Cd1	142.55 (13)	O1—C1—N1	124.6 (3)
C12—N12—C11	117.4 (3)	O1—C1—H1	117.7
C12—N12—Cd1	143.1 (2)	N1—C1—H1	117.7
C11—N12—Cd1	99.53 (18)	N1—C2—H2A	109.5
C14—N13—C11	115.3 (3)	N1—C2—H2B	109.5
C18—N14—H14A	122 (3)	H2A—C2—H2B	109.5
C18—N14—H14B	123 (3)	N1—C2—H2C	109.5
H14A—N14—H14B	111 (3)	H2A—C2—H2C	109.5
C21—N21—S21	122.2 (2)	H2B—C2—H2C	109.5
C21—N21—Cd1	111.16 (18)	N1—C3—H3A	109.5
S21—N21—Cd1	123.05 (13)	N1—C3—H3B	109.5
C22—N22—C21	116.9 (3)	H3A—C3—H3B	109.5
C22—N22—Cd1	154.3 (2)	N1—C3—H3C	109.5
C21—N22—Cd1	83.03 (17)	H3A—C3—H3C	109.5
C24—N23—C21	115.7 (3)	H3B—C3—H3C	109.5
C28—N24—H24A	120 (3)	O2—C4—N2	124.6 (3)
C28—N24—H24B	116 (2)	O2—C4—H4	117.7
H24A—N24—H24B	110 (3)	N2—C4—H4	117.7
N13—C11—N12	124.9 (3)	N2—C5—H5A	109.5
N13—C11—N11	124.3 (3)	N2—C5—H5B	109.5
N12—C11—N11	110.7 (2)	H5A—C5—H5B	109.5
N12—C12—C13	121.7 (3)	N2—C5—H5C	109.5
N12—C12—H12	119.1	H5A—C5—H5C	109.5
C13—C12—H12	119.1	H5B—C5—H5C	109.5
C12—C13—C14	116.7 (3)	N2—C6—H6A	109.5
C12—C13—H13	121.6	N2—C6—H6B	109.5
C14—C13—H13	121.6	H6A—C6—H6B	109.5
N13—C14—C13	124.0 (3)	N2—C6—H6C	109.5
N13—C14—H14	118.0	H6A—C6—H6C	109.5
C13—C14—H14	118.0	H6B—C6—H6C	109.5
C16—C15—C20	119.8 (3)	C1—N1—C3	121.1 (3)
C16—C15—S11	120.8 (2)	C1—N1—C2	121.3 (3)
C20—C15—S11	118.8 (2)	C3—N1—C2	117.7 (3)
C17—C16—C15	120.1 (3)	C4—N2—C5	121.2 (3)
C17—C16—H16	119.9	C4—N2—C6	120.3 (3)
C15—C16—H16	119.9	C5—N2—C6	118.5 (3)
C16—C17—C18	120.9 (3)	C1—O1—Cd1	122.91 (19)
C16—C17—H17	119.5	C4—O2—Cd1	119.23 (19)
C18—C17—H17	119.5	Cd1—O3—H3D	120 (3)
N14—C18—C17	121.1 (3)	Cd1—O3—H3E	103 (3)
N14—C18—C19	120.4 (3)	H3D—O3—H3E	108 (3)
C17—C18—C19	118.5 (3)	H4A—O4—H4B	108 (7)
C20—C19—C18	120.7 (3)		
			
O12—S11—N11—C11	−57.5 (3)	C11—N12—C12—C13	1.2 (4)
O11—S11—N11—C11	177.3 (2)	Cd1—N12—C12—C13	−179.2 (2)
C15—S11—N11—C11	65.2 (3)	N12—C12—C13—C14	−0.3 (4)
O12—S11—N11—Cd1	127.7 (2)	C11—N13—C14—C13	0.7 (4)
O11—S11—N11—Cd1	2.5 (3)	C12—C13—C14—N13	−0.8 (5)
C15—S11—N11—Cd1	−109.6 (2)	O12—S11—C15—C16	−20.0 (3)
N21—Cd1—N11—C11	22.0 (4)	O11—S11—C15—C16	102.3 (2)
O1—Cd1—N11—C11	−95.87 (17)	N11—S11—C15—C16	−146.8 (2)
O3—Cd1—N11—C11	−175.93 (18)	O12—S11—C15—C20	168.3 (2)
O2—Cd1—N11—C11	85.64 (17)	O11—S11—C15—C20	−69.5 (2)
N12—Cd1—N11—C11	−0.85 (15)	N11—S11—C15—C20	41.5 (3)
N22—Cd1—N11—C11	138.50 (15)	C20—C15—C16—C17	1.2 (4)
N21—Cd1—N11—S11	−162.3 (3)	S11—C15—C16—C17	−170.5 (2)
O1—Cd1—N11—S11	79.8 (2)	C15—C16—C17—C18	−1.2 (5)
O3—Cd1—N11—S11	−0.3 (2)	C16—C17—C18—N14	178.5 (3)
O2—Cd1—N11—S11	−98.7 (2)	C16—C17—C18—C19	−0.3 (4)
N12—Cd1—N11—S11	174.8 (3)	N14—C18—C19—C20	−176.9 (3)
N22—Cd1—N11—S11	−45.9 (3)	C17—C18—C19—C20	2.0 (4)
N21—Cd1—N12—C12	7.3 (3)	C18—C19—C20—C15	−2.1 (4)
O1—Cd1—N12—C12	−95.2 (3)	C16—C15—C20—C19	0.5 (4)
O3—Cd1—N12—C12	−171.8 (3)	S11—C15—C20—C19	172.3 (2)
O2—Cd1—N12—C12	93.9 (3)	C24—N23—C21—N22	−2.8 (4)
N11—Cd1—N12—C12	−178.8 (4)	C24—N23—C21—N21	175.9 (3)
N22—Cd1—N12—C12	55.8 (4)	C22—N22—C21—N23	2.3 (4)
N21—Cd1—N12—C11	−173.05 (16)	Cd1—N22—C21—N23	165.3 (3)
O1—Cd1—N12—C11	84.45 (17)	C22—N22—C21—N21	−176.5 (3)
O3—Cd1—N12—C11	7.8 (2)	Cd1—N22—C21—N21	−13.5 (2)
O2—Cd1—N12—C11	−86.48 (17)	S21—N21—C21—N23	−1.0 (4)
N11—Cd1—N12—C11	0.87 (15)	Cd1—N21—C21—N23	−160.3 (2)
N22—Cd1—N12—C11	−124.55 (18)	S21—N21—C21—N22	177.9 (2)
O22—S21—N21—C21	−171.0 (2)	Cd1—N21—C21—N22	18.6 (3)
O21—S21—N21—C21	63.2 (3)	C21—N22—C22—C23	−0.2 (4)
C25—S21—N21—C21	−54.7 (3)	Cd1—N22—C22—C23	−138.0 (4)
O22—S21—N21—Cd1	−14.09 (18)	N22—C22—C23—C24	−1.3 (5)
O21—S21—N21—Cd1	−139.98 (14)	C21—N23—C24—C23	1.1 (4)
C25—S21—N21—Cd1	102.20 (16)	C22—C23—C24—N23	0.8 (5)
O1—Cd1—N21—C21	−125.84 (18)	O22—S21—C25—C26	−109.1 (3)
O3—Cd1—N21—C21	−42.6 (2)	O21—S21—C25—C26	16.0 (3)
O2—Cd1—N21—C21	54.96 (18)	N21—S21—C25—C26	137.7 (2)
N12—Cd1—N21—C21	138.07 (17)	O22—S21—C25—C30	73.6 (3)
N11—Cd1—N21—C21	118.0 (3)	O21—S21—C25—C30	−161.2 (2)
N22—Cd1—N21—C21	−10.30 (15)	N21—S21—C25—C30	−39.5 (3)
O1—Cd1—N21—S21	75.04 (16)	C30—C25—C26—C27	−0.1 (5)
O3—Cd1—N21—S21	158.30 (13)	S21—C25—C26—C27	−177.3 (2)
O2—Cd1—N21—S21	−104.16 (15)	C25—C26—C27—C28	0.5 (5)
N12—Cd1—N21—S21	−21.05 (18)	C26—C27—C28—N24	177.1 (3)
N11—Cd1—N21—S21	−41.1 (4)	C26—C27—C28—C29	−1.2 (5)
N22—Cd1—N21—S21	−169.4 (2)	N24—C28—C29—C30	−176.9 (3)
N21—Cd1—N22—C22	152.7 (5)	C27—C28—C29—C30	1.5 (5)
O1—Cd1—N22—C22	−125.0 (4)	C28—C29—C30—C25	−1.0 (5)
O3—Cd1—N22—C22	−58.7 (4)	C26—C25—C30—C29	0.3 (5)
O2—Cd1—N22—C22	47.2 (4)	S21—C25—C30—C29	177.6 (2)
N12—Cd1—N22—C22	87.8 (5)	O1—C1—N1—C3	176.4 (3)
N11—Cd1—N22—C22	−10.1 (5)	O1—C1—N1—C2	−2.7 (5)
N21—Cd1—N22—C21	9.83 (15)	O2—C4—N2—C5	−1.9 (5)
O1—Cd1—N22—C21	92.09 (16)	O2—C4—N2—C6	177.5 (3)
O3—Cd1—N22—C21	158.45 (17)	N1—C1—O1—Cd1	165.9 (2)
O2—Cd1—N22—C21	−95.66 (16)	N21—Cd1—O1—C1	1.1 (2)
N12—Cd1—N22—C21	−55.1 (2)	O3—Cd1—O1—C1	−111.2 (2)
N11—Cd1—N22—C21	−153.00 (15)	O2—Cd1—O1—C1	176.5 (4)
C14—N13—C11—N12	0.3 (4)	N12—Cd1—O1—C1	112.7 (2)
C14—N13—C11—N11	−179.3 (3)	N11—Cd1—O1—C1	167.8 (2)
C12—N12—C11—N13	−1.3 (4)	N22—Cd1—O1—C1	−50.2 (2)
Cd1—N12—C11—N13	179.0 (2)	N2—C4—O2—Cd1	138.8 (3)
C12—N12—C11—N11	178.4 (2)	N21—Cd1—O2—C4	−113.0 (2)
Cd1—N12—C11—N11	−1.4 (2)	O1—Cd1—O2—C4	71.6 (5)
S11—N11—C11—N13	4.1 (4)	O3—Cd1—O2—C4	0.8 (2)
Cd1—N11—C11—N13	−179.1 (3)	N12—Cd1—O2—C4	135.9 (2)
S11—N11—C11—N12	−175.53 (19)	N11—Cd1—O2—C4	80.2 (2)
Cd1—N11—C11—N12	1.3 (2)	N22—Cd1—O2—C4	−64.8 (2)

### Hydrogen-bond geometry (Å, °)

**Table:**

*D*—H···*A*	*D*—H	H···*A*	*D*···*A*	*D*—H···*A*
N14—H14B···O12^i^	0.82 (2)	2.39 (3)	3.104 (4)	146 (4)
N14—H14A···O21^ii^	0.81 (2)	2.47 (2)	3.267 (4)	167 (4)
N24—H24B···O4^iii^	0.84 (2)	2.27 (2)	3.061 (4)	157 (3)
N24—H24A···O3^iii^	0.79 (2)	2.52 (2)	3.272 (4)	160 (3)
O3—H3E···O11	0.79 (2)	1.97 (2)	2.754 (3)	172 (4)
O3—H3D···O11^iv^	0.81 (2)	2.04 (3)	2.810 (3)	159 (3)
O4—H4B···O21	0.85 (3)	2.02 (4)	2.855 (3)	166 (12)
O4—H4A···N23^v^	0.85 (3)	2.30 (5)	3.051 (4)	148 (7)

Symmetry codes: (i) −*x*+1/2, *y*+1/2, −*z*−1/2; (ii) −*x*, −*y*+1, −*z*; (iii) *x*, *y*−1, *z*; (iv) −*x*+1, −*y*+1, −*z*; (v) −*x*+1/2, *y*+1/2, −*z*+1/2.
